# CRF Receptor Antagonist Astressin-B Reverses and Prevents Alopecia in CRF Over-Expressing Mice

**DOI:** 10.1371/journal.pone.0016377

**Published:** 2011-02-16

**Authors:** Lixin Wang, Mulugeta Million, Jean Rivier, Catherine Rivier, Noah Craft, Mary P. Stenzel-Poore, Yvette Taché

**Affiliations:** 1 Division of Digestive Diseases, Department of Medicine, CURE and Center for Neurobiological Stress, David Geffen School of Medicine at University of California Los Angeles, VA Greater Los Angeles Healthcare System, Los Angeles, California, United States of America; 2 Clayton Foundation Laboratories for Peptide Biology, Salk Institute for Biological Studies, San Diego, California, United States of America; 3 Divisions of Dermatology and Infectious Diseases, Department of Medicine, Los Angeles Biomedical Research Institute at Harbor-UCLA Medical Center, David Geffen School of Medicine at University of California Los Angeles, Torrance, California, United States of America; 4 Department of Molecular Microbiology and Immunology, Oregon Health & Sciences University, Portland, Oregon, United States of America; Texas A&M University, United States of America

## Abstract

Corticotropin-releasing factor (CRF) signaling pathways are involved in the stress response, and there is growing evidence supporting hair growth inhibition of murine hair follicle *in vivo* upon stress exposure. We investigated whether the blockade of CRF receptors influences the development of hair loss in CRF over-expressing (OE)-mice that display phenotypes of Cushing's syndrome and chronic stress, including alopecia. The non-selective CRF receptors antagonist, astressin-B (5 µg/mouse) injected peripherally once a day for 5 days in 4–9 months old CRF-OE alopecic mice induced pigmentation and hair re-growth that was largely retained for over 4 months. In young CRF-OE mice, astressin-B prevented the development of alopecia that occurred in saline-treated mice. Histological examination indicated that alopecic CRF-OE mice had hair follicle atrophy and that astressin-B revived the hair follicle from the telogen to anagen phase. However, astressin-B did not show any effect on the elevated plasma corticosterone levels and the increased weights of adrenal glands and visceral fat in CRF-OE mice. The selective CRF_2_ receptor antagonist, astressin_2_-B had moderate effect on pigmentation, but not on hair re-growth. The commercial drug for alopecia, minoxidil only showed partial effect on hair re-growth. These data support the existence of a key molecular switching mechanism triggered by blocking peripheral CRF receptors with an antagonist to reset hair growth in a mouse model of alopecia associated with chronic stress.

## Introduction

More than half a century ago, Hans Selye, the father of the stress concept in biology, stated that “an intense psychic shock may also exert pronounced effects on the hair, e.g., graying and generalized loss of hair” [1]. Subsequent cumulative experimental and clinical evidence indicates indeed, that chronic stress exerts a profound inhibitory effect on hair growth [2]–[5]. Corticotropin-releasing factor (CRF), adrenocorticotropic hormone (ACTH) and glucocorticoids not only are key components of the endocrine and neuroimmune responses to stress but also they interrupt hair follicle growth cycle in humans and mice [2], [3], [6], [7]. In cultured human scalp hair follicles, CRF up-regulates transcription of pro-opiomelanocortin (POMC) and immunoreactivity of ACTH and α-melanocyte-stimulating hormone (MSH), and increases cortisol secretion [5]. Slominski et al. [8], [9] have also shown that CRF, urocortin 1 and CRF receptor subtypes 1 and 2 (CRF_1_ and CRF_2_) are expressed in the normal skin and cycling hair follicles of humans and mice.

Mice that over-express CRF (CRF-OE) have been characterized as a model of chronic stress that captures phenotypes of behavioral, endocrine, immunological, autonomic and visceral alterations beside Cushing's syndrome manifestations [10]–[16]. While a number of mouse mutants generated by targeting specific pathways involving hair follicle cycle resulted in nude mice or models of inflammatory alopecia [4], [17], [18], the CRF-OE mouse has not been examined so far as a model relevant to chronic stress-induced alopecia, despite an initial report that CRF-OE mice develop bilateral symmetric hair loss in adulthood [11].

Based on existing evidence that chronic stress impairs hair growth and that major components of the CRF system are expressed in the mouse and human skin [9], [19], we investigated the ability of CRF receptor antagonists to influence hair loss/re-growth in CRF-OE mice. We assessed whether blocking CRF receptors by short-term peripheral treatment with the long acting peptide CRF_1_/CRF_2_ receptors antagonist, astressin-B [20] would induce hair re-growth and pigmentation in adult alopecic CRF-OE mice and prevent the development of alopecia in young CRF-OE mice. We also investigated the specificity of the CRF antagonist action on hair growth or whether it would also affect elevated plasma corticosterone levels and other Cushing-like phenotypes (such as hypertrophy of the adrenal glands and increased adipose deposits) [11]. Lastly, we tested under similar conditions whether the selective CRF_1_ receptor non peptide antagonist, NBI 27914 [21], the selective CRF_2_ receptor peptide antagonist, astressin_2_-B [22] or a commercial drug, minoxidil [23] exert effects on hair growth and pigmentation.

## Results

### The non-selective CRF_1_/CRF_2_ antagonist, astressin-B injected intraperitoneally (ip) or subcutaneously (sc) reverses alopecia in CRF-OE mice

**Figure 1:**
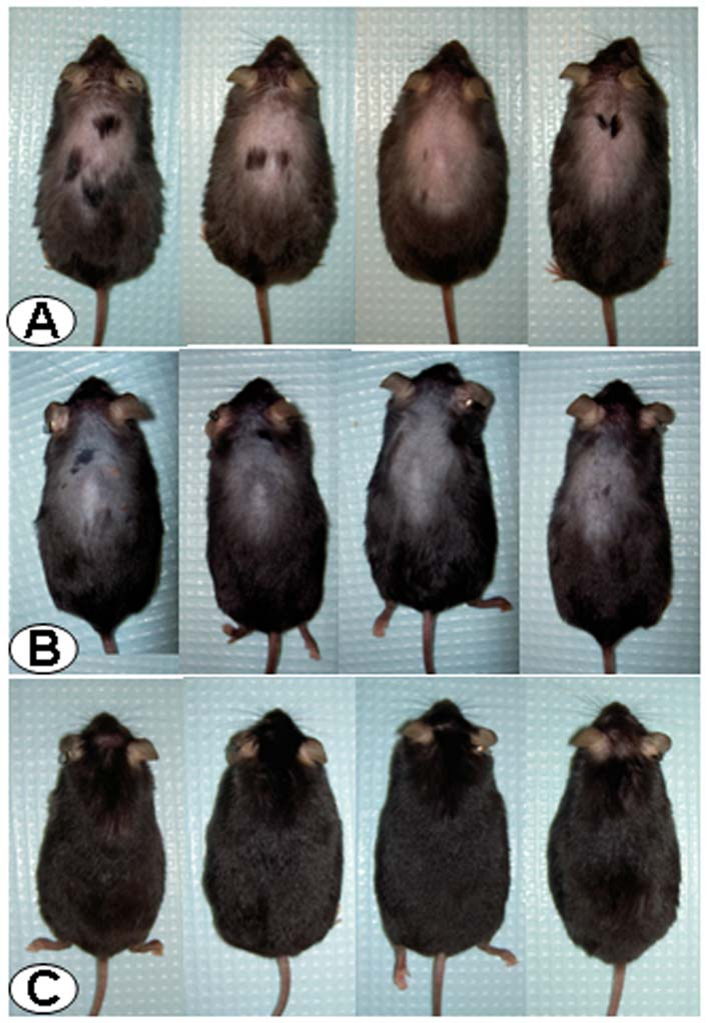
The CRF_1_/CRF_2_ receptor antagonist, astressin-B, injected intraperitoneally (ip) in CRF-OE mice with fully developed alopecia induces hair growth and pigmentation. Photographs: Row A: Male CRF-OE mice (4 months old) injected ip once daily for 5 consecutive days with saline at 3 days after the last injection and Row B: astressin-B (5 µg/mouse) at 3 days after the last ip injection, and Row C: the same mice as in the middle panel Row B at 4 weeks after the last ip injection.

**Figure 2:**
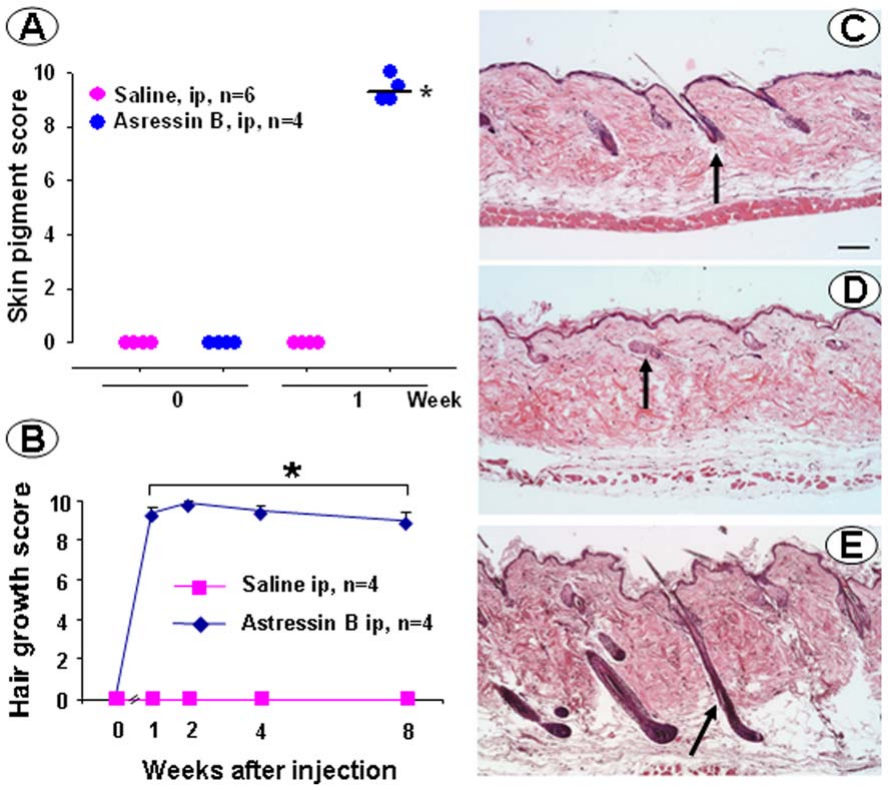
Skin pigment score (A), hair growth score (B) and skin photomicrographs (C–E) of male CRF-OE mice (7 months old) injected ip once a day for 5 consecutive days with astressin-B (5 µg/mouse) or saline. Data are individual and median in A and mean ± SEM in B, p<0.05 vs before the ip injection. Photomicrographs show the hair follicle morphology (scale 100 µm) in the back skin of male wild type mice (C) and CRF-OE mice at 2 weeks after the last ip injection of saline (D) or astressin-B (E). Arrows indicate a hair follicle in each panel.

**Figure 3:**
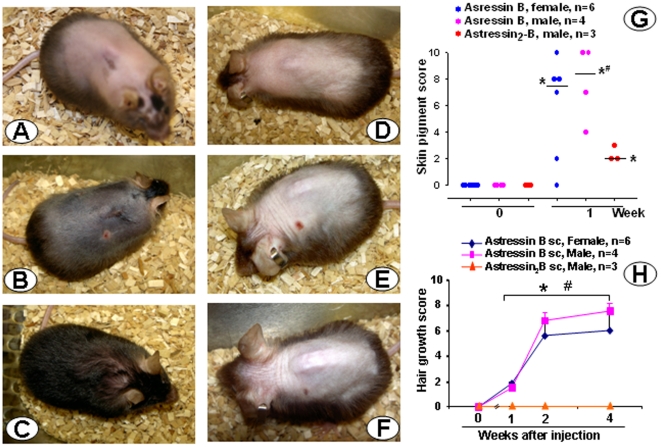
Subcutaneous (sc) injection of CRF_1_/CRF_2_ receptor antagonist, astressin-B induces pigmentation and hair growth in CRF-OE mice with alopecia. Groups of 6–9 month old male or female CRF-OE mice were injected sc once daily for 5 consecutive days either with astressin-B (5 µg/mouse) or astressin_2_-B (5 µg/mouse). A–C: A fully alopecic female CRF-OE mouse before (A) and at 1 (B) and 4 weeks (C, with 90% hair coverage), following the last sc astressin-B injection. D–F: A fully alopecic male mouse before (D) and at 1 (E) or 4 weeks (F) after the last sc injection of astressin_2_-B, with no hair re-growth. G: Scores of skin pigmentation before the start of injection and 1 week after the last sc injection; H: time course of hair growth score before and up to 4 weeks after last sc injection. Data are individual and median in G and mean ± SEM in H. * p<0.05 vs week 0 (G and H); # p<0.05 vs astressin_2_-B at the corresponding week (G) and (H).

Male and female CRF-OE mice develop alopecia when they are older than 4 months. Saline injected ip in male CRF-OE mice did not have any effect on the alopecia: the skin color remained pink and no hair grew throughout the monitoring period (Figs. 1A and 2A, B). By contrast, the CRF_1_/CRF_2_ receptor antagonist, astressin-B injected ip at 5 µg/mouse once a day for 5 consecutive days resulted in the development of dark pigment on the initially pink alopecic skin within 3 days after the last injection in 4 months old male CRF-OE mice (Fig. 1B). Simultaneously, as the pigment increased to a maximal response within 7–10 days (Fig. 2A), hairs sprouted out and grew to full length with 95–100% of hair coverage in 2 weeks (Figs. 1C and 2B). The re-grown hair was retained for the following 8 weeks (Fig. 2B), and then largely maintained up to 4 months post injection when mice were euthanized (data not shown). Similarly, astressin-B (5 µg/mouse) injected sc once a day for 5 days induced skin pigment within one week after the last injection in 80% of the 4–9 months old alopecic female and male CRF-OE mice (Fig 3B, G). Mice regained 50–90% hair coverage at 2–4 weeks post treatment (Fig. 3A–C, H).

In contrast, sc injection of astressin_2_-B (5 µg/mouse once a day for 5 consecutive days) did not induce hair growth in CRF-OE mice (Fig. 3E–H) although a low grade but significant pigmentation occurred (Fig. 3G). Likewise, the CRF_1_ antagonist, NBI 27914 (0.5 mg/mouse) injected sc twice a day for 5 days in 5 months old female CRF-OE mice did not induce any hair re-growth (data not shown).

### Skin morphological changes in CRF-OE mice injected intraperitoneally with astressin-B

Histological assessment showed that the hair follicles in the alopecic skin from CRF-OE mice (7 months old, male) injected ip with saline (Fig. 2D) had aberrant morphology compared to that in the WT littermates (Fig. 2C). The length of the hair follicle in CRF-OE mice was significantly shorter than that in the WT (0.37±0.03 vs. 0.54±0.03 mm; n = 5/group; P<0.05). In ip astressin-B-treated CRF-OE mice, hair follicle reformation was seen 2 weeks after the treatment (Fig. 2E) and the hair follicles were changed from the telogen to anagen phase (Fig. 2D, E). The average length of the hair follicles was 0.42±0.05 mm (n = 5) and no longer significantly different from those in WT littermates.

### Lack of effect of intraperitoneal astressin-B on other features of Cushing's syndrome in CRF-OE mice

**Figure 4:**
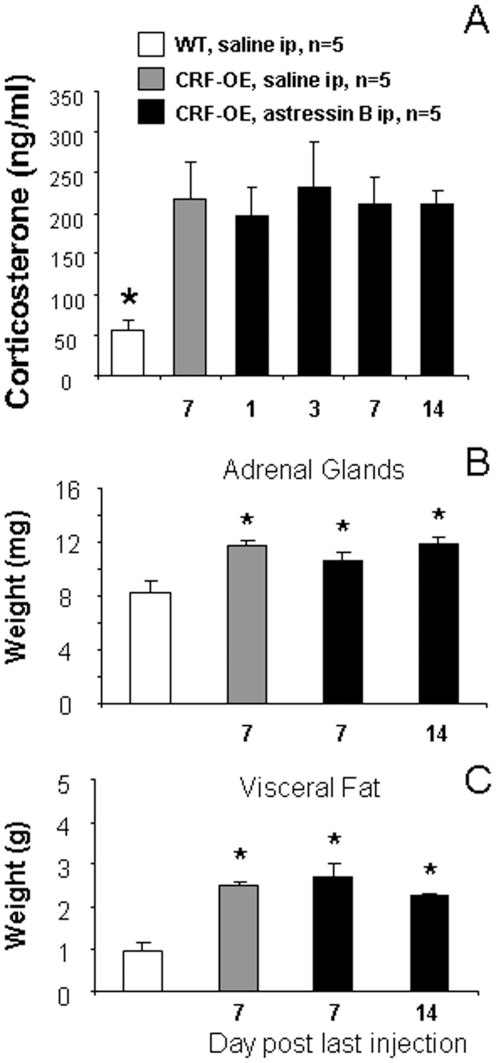
Astressin-B injected intraperitoneally (ip) does not influence plasma corticosterone (A), adrenal gland (B) and visceral fat weights (C) in female CRF-OE mice (4–7 months old). Each bar represents the mean ± SEM (n = 5/group or time point). * p<0.05 vs WT.

CRF-OE mice (4–7 months old, female) had a significant 3.9 times higher plasma corticosterone levels than WT littermates as assessed at day 7 after the last ip saline injection (Fig. 4A). Astressin-B (5 µg/mouse) injected ip for 5 days reversed alopecia but did not modify the elevated plasma corticosterone levels in CRF-OE mice at any of the days monitored, i.e, days 1, 3, 7 and 14 after the last ip astressin-B injection (Fig. 4A). In addition, the weights of adrenal glands and visceral fat were significantly greater in CRF-OE than in WT mice, and unchanged by ip astressin-B injections as monitored on days 7 and 14 after the last peptide injection (Fig. 4B, C).

### Astressin-B given subcutaneously in young CRF-OE mice prevents development of alopecia

**Figure 5:**
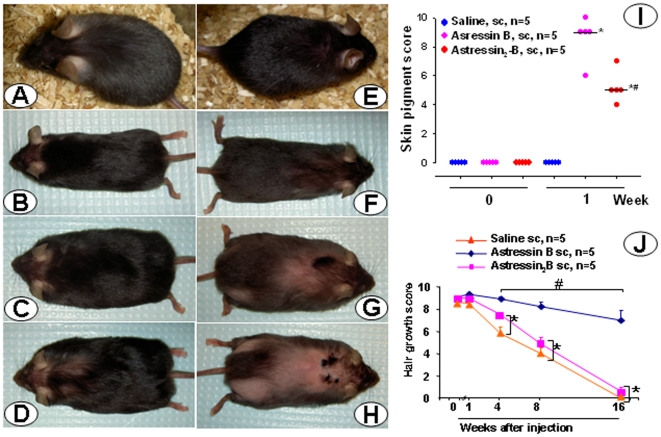
The CRF_1_/CRF_2_ receptor antagonist, astressin-B injected subcutaneously (sc) induces pigmentation and prevents hair loss in young CRF-OE mice with not yet developed alopecia. Photos: representative CRF-OE female mice before injection (A, E) and the same mice at 4, 8 and 16 weeks after astressin-B (B–C) or astressin_2_-B (F–H) injected sc once daily for 5 consecutive days at 5 µg/mice. Graph I: Individual and median values of skin pigmentation scores before (week 0) and 1 week after the treatment. *: p<0.05 vs groups in week 0 and saline in week 1; #: P<0.05 vs astressin_2_-B, Graph J: scores of hair growth before (week 0) and 1, 4, 8 and 16 weeks after the treatment. Each point represents the mean ± SEM. *: p<0.05 vs week 0, before and after; #: p<0.05 vs. astressin_2_-B or saline at the corresponding weeks.

Young CRF-OE mice (7–8 weeks of age, female) with little hair loss injected sc with saline developed alopecia in 70–100% area of the back during the 8–16 weeks after injection (Fig. 5J). By contrast, young CRF-OE mice injected sc with astressin-B (5 µg/mouse/day for 5 days) displayed skin pigmentation within one week after the last injection (Fig. 5I), did not have hair loss from the back for the following 2 months and kept on average 70% of the hair at the end of the 4 months observation period post injection (Fig. 5A–D, J). On the other hand, sc astressin_2_-B-injected CRF-OE mice showed a moderate induction of skin pigment during the first week post injection (Fig. 5I), but continued to lose hair similarly as the saline-treated mice (Fig. 5.E–H, J).

### Effect of minoxidil given subcutaneously to CRF-OE mice with alopecia

**Figure 6:**
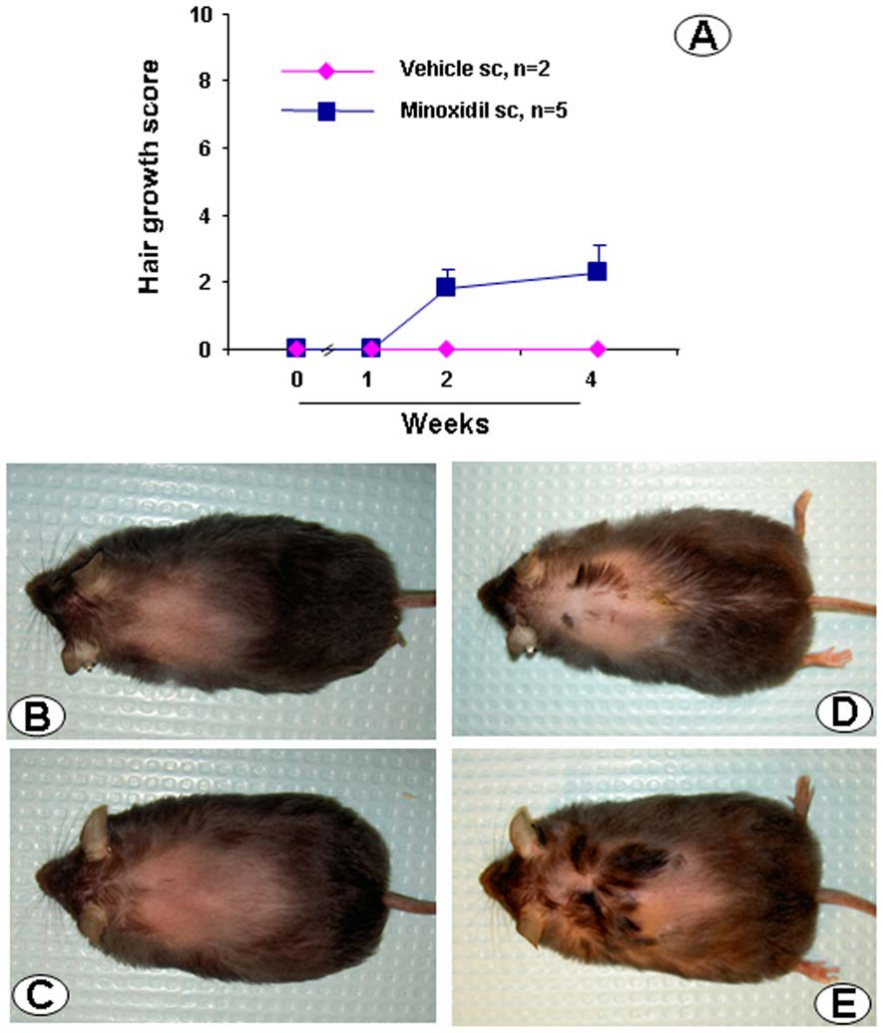
Effects of 1% minoxidil sulfate injected subcutaneously (sc) on alopecia in CRF-OE mice. Groups of 5 months old CRF-OE mice were injected sc with either vehicle or minoxidil sulfate (1 mg/mouse) for 10 days. Hair growth score was monitored at 1, 2 and 4 weeks after the last injection. A: scores of hair growth before (week 0) and at weeks 2 and 4 after the treatment. Each point represents the mean ± SEM, n = 5/group. B–E: representative photos of CRF-OE mice before (B &D) and at 4 weeks after the last injection of vehicle (C) or minoxidil (F).

The administration of 1% minoxidil sulfate solution (0.1 ml/mouse, sc) once a day for 10 days in 5 months old female alopecic CRF-OE mice induced patches of pigment 2 weeks after the last injection in 3 out of the 4 treated CRF-OE mice and a moderate hair growth in the pigmented patches was observed at 2 and 4 weeks after the last injection while vehicle injection had no effect (Fig. 6).

## Discussion

The present experiments demonstrate that, in the CRF-OE mice with alopecia, blockade of both CRF_1_ and CRF_2_ receptors with intraperitoneal or subcutaneous injection of the peptide CRF_1_ and CRF_2_ receptor antagonist, astressin-B [20] induces a robust skin pigmentation and hair re-growth. In addition, in young not yet alopecic CRF-OE mice, astressin-B prevents hair loss. Remarkably, the hair re-growth was observed with as little as 5 daily single injections of 5 µg/mice. This study also provides evidence that CRF-OE mice display features of a relevant model to study alopecia.

Astressin-B action may involve blockade of both CRF_1_ and CRF_2_ that are expressed in murine skin including hair follicles [8], [24]. This is supported by the data that neither the long acting selective CRF_2_ antagonist, astressin_2_-B [22] nor the selective CRF_1_ antagonist, NBI 27914 [21] given alone had effect on hair growth when administered subcutaneously at doses blocking exogenous CRF or urocortin 1 actions [22], [25]. Similarly, in young CRF-OE mice, subcutaneous injection of astressin-B prevented the development of alopecia in the subsequent several weeks/months monitoring time. However, saline and astessin_2_-B treated CRF-OE mice developed full alopecia although selective blockade of CRF_2_ receptors by astessin_2_-B, resulted in mild and brief increased pigmentation. Additional research is needed to assess the respective role of CRF receptor subtypes on hair re-growth and pigmentation. In particular whether different dosing regimens of CRF_1_- or CRF_2_-selective antagonists induce measurable effects on hair growth and pigmentation as well as identification of molecule(s) and cells on which astressin-B acts to trigger its effects require further studies. Regardless, the present data provide the first evidence of the potency and efficacy of astressin-B injected peripherally to induce a long term reversal or prevention of alopecia in CRF-OE mice. These murine data are also in line with the effect of CRF agonists and antagonists on human hair follicle elongation [5].

Because astressin-B was injected peripherally in CRF-OE mice, it is possible that the observed effect may have resulted not only from a local action but also through decreasing the deleterious effects that chronic systemic activation of the pituitary and adrenal secretion may have on skin pigmentation and hair re-growth [2], [3]. Indeed, adrenalectomy was shown to reverse alopecia in CRF-OE mice [11]. However, in the present study we did not detect changes in the elevated corticosterone plasma levels in CRF-OE mice at each time point monitored on days 1, 3, 7 and 14 after the end of astressin-B treatment. These data are consistent with our previous pharmacokinetic studies showing that under chronic stimulation of the hypothalamic-pituitary axis, astressin-B injected peripherally blocked the elevated ACTH plasma levels for 12 h while at 24 h post injection values are back to those of vehicle [20]. In addition, the CRF antagonist was given as a single injection once a day for only 5 days and yet the re-grown hair in most of the mice was maintained for up to several months. The prevention of hair loss was still remarkable 3 to 4 months post treatment. Therefore, although the ablation of the adrenals reversed the alopecia in CRF-OE mice [11], the fact that astressin-B, at the regimen used, exerted such dramatic effect against the alopecia without affecting plasma corticosterone levels rules out the possibility that peripheral injection of astressin-B exerts a persistent inhibition of the ACTH-corticosterone cascade for such a long period. This is further supported by the demonstration that although the short term astressin-B treatment induced hair regrowth, it did not affect other Cushing syndrome-like manifestations such as increased weights of adrenal glands and abdominal fat deposits in CRF-OE.

One of the striking effects of astressin-B injected ip or sc was the strong induction of skin/hair pigment within a few days following the 5 days treatment regimen. Likewise, sc injection of the CRF_2_ receptor antagonist, astressin_2_-B induced a partial induction of skin pigmentation and more so in young than alopecic CRF-OE mice at one week after treatment. Hair pigment is under the control of complex systems including neuroendocrine, neurotrophins, melanocortin receptor signaling, melanin synthesis, and melanin transport and incorporation to hair shaft keratinocytes [26], [27]. Several factors, including CRF, POMC, ACTH and corticoids, play a role in turning on or off the activity of hair follicle melanocytes [27], [28]. Interestingly, cutaneous melanocytes respond to environmental stress such as UVB by the production of CRF [29]. CRF_1_ and CRF_2_ receptors are differentially expressed and exert distinct function in human and murine hair follicle pigmentary units [6], [8], [30], [31]. Urocortin 1, a mammalian member of CRF peptide family with higher affinity to CRF_2_ receptor than CRF down-regulates melanocyte differentiation phenotype [30]. The fact that pigmentation occurred together with the induction of hair growth in the astressin-B-treated mice is in agreement with the data that melanogenesis is strictly coupled to anagen hair growth phase [32]. Thus, CRF overexpression may suppress melanocyte activity, whereas blockade of the CRF receptors by astressin-B turns on their activity to cause skin/hair pigmentation. By contrast, inhibition of CRF_2_ receptors by astressin_2_-B only partially stimulates activity.

Although a tremendous stride has been made in the understanding of underlying cellular mechanisms of hair growth and alopecia, there is a paucity of experimental models, particularly those pertaining to chronic stress-related hair loss and their remedy. The fact that the chronically stressed CRF-OE mice become alopecic in adulthood is reminiscent of human hair loss associated with stress [33]. Alopecia in the CRF-OE mice affects primarily, although is not limited to, the back. Additionally, it is responsive to treatments such as a non-selective, long acting CRF receptor antagonist, and to a lesser extent to minoxidil, one of the few federally approved clinical treatment of alopecia [21]. These findings suggest that the alopecia in CRF-OE mice share some features as seen in humans. The CRF-OE mice with sustained elevated levels of CRF and corticosterone and hair loss as seen in the present and prior studies [11] have good face and construct validity as a relevant model for alopecia and therefore provide a unique opportunity to unravel pathways in chronic stress-related hair loss.

Collectively, we have shown that peripheral blockade of the CRF_1_ and CRF_2_ receptors with astressin-B injected peripherally either subcutaneous or intraperitoneally for five consecutive days at a dose as low as 5 µg/CRF-OE mouse activates skin pigmentation and hair growth in alopecic mice and prevents hair loss in young CRF-OE mice. Temporary blockade of the CRF receptors could thus be a breakthrough therapy for alopecia particularly for patients in acute (chemotherapy, traumatic stressful events) or chronic stress setting. Moreover, the present findings indicate that CRF-OE mice could be a relevant model to advance our understanding of chronic stress-related hair loss.

## Materials and Methods

### Ethics Statement

The study involved the use of rodents. All experiments were performed in accordance to relevant international and national guidelines for use of laboratory animals. All procedures were approved by the Veteran Affairs Greater Los Angeles Healthcare System Animal Research Committees (Protocols 04012-06, 9906-820 and 11084-03).

### Mice

Male and female CRF-OE and wild type (WT) littermates were derived from the transgenic line generated on C57BL/6 X SJL background that was backcrossed over 10 generations onto C57BL/6 as detailed previously using a chimeric CRF transgene comprising the methallothionein-1 promoter driving the rat CRF gene [11]. The mice were housed under controlled temperature (21–23°C), humidity (30–35%) and light cycle (6:00 AM to 6:00 PM) and maintained on a standard rodent diet (Prolab RMH 2500, LabDiet, PMI Nutrition, Brentwood, MO).

### Substances

Astressin-B and astressin_2_-B (Peptide Biology Laboratory, The Salk Institute, La Jolla, CA) were synthesized using the solid phase approach and the Boc-strategy as previously described [20], [22] and stored in powder form at −80°C. Immediately before the experiments, peptides were dissolved in sterile water (pH 7.0). NBI-27914 (Tocris, Ellisville MO) was dissolved in ethanol, Tween 80 and saline [1∶0.5∶8.5 (v/v), pH 5.0]. Minoxidil sulfate salt (Sigma-Aldrich Co, St Louis, MN) was dissolved in 1∶2∶7 (v/v) ethanol, polyethylene glycol and saline, respectively, pH 5.0.

### Measurement of hair growth and pigmentation

The monitoring of hair growth was essentially as described by Vegesna et al. [34]. Thus scores were made visually as well as using photographs of each mouse at a weekly interval. Hair-growth was observed in a dorsal region on the back of CRF-OE mice that either fully developed alopecia (age ≥4 months) or not yet developed alopecia (≤8 weeks). The area was defined anteriorly by a line joining the front base of the ears, posteriorly by a line along the front of the pelvic girdle and laterally by the lines projecting caudally from each ear base. A zero score corresponded to a no change in the amount of hair in the test alopecic area and the score of 10 to full hair growth in the entire alopecic area. In brief, the percent area that is covered by hair in the above-delimited area is scored between 0–10 to correspond to hair coverage of 0–100% of the test region.

Pigmentation of the skin in the test region was also measured because, in C57BL/6 mice, the skin darkens at the onset of hair growth. A pigmentation score from 0–10 was given to each mouse in the test region: 0: bright pink skin color/pigment; 1–5: increasing intensity of a pale grayish color/pigment of skin; 6–9: increasing but non-confluent darker color/pigment of skin; 10: black pigmentation over the total test area. Pigmentation was assessed one week after the final injection because changes (if any) in pigmentation are substantially complete by that time.

### Skin histology

CRF-OE and WT mice (male, 7 months old) injected ip with saline or astressin-B (5 µg/mouse) for 5 consecutive days were euthanized 2 weeks after the last injection with an overdose of sodium pentobarbital (5 mg/0.1 ml/mouse, ip; Nembutal®, Abbott Laboratories, Chicago, IL, USA) and skin samples were obtained from the back (the alopecic area) and fixed in 4% paraformaldehyde. Paraffin-embedded sections were processed for hematoxylin/eosin staining and examined at light microscopy (Zeiss Axioskp 2). Photomicrographs were acquired by a digital camera (Hamamatsu, Bridgewater, NJ) using the image acquisition system SimplePCI (Hamamatsu Corporation, Sewickley, PA). The length of hair follicle was measured using NIH Image J version 1.42.

### Plasma corticosterone levels, adrenal gland and visceral fat weight determinations

CRF-OE mice and WT littermates (female, 4–7 months old) were injected ip (0.1 ml) with astressin-B (5 µg/mouse) or saline daily for 5 days and euthanized 7 days post last saline injection or at day 1, 3, 7 or 14 after the last injection of astressin-B. Blood was collected from the heart in less than a minute from mice anesthetized with pentobarbital sodium (5 mg/0.1 ml/mouse, ip). Plasma was immediately isolated and stored at −80°C until assay. Plasma corticosterone levels were measured by Immunochem I-125 corticosterone RIA kit (ICN Biomedical Inc., Costa Mesa, CA). The assay was previously validated in mice [35], [36]. At day 7 post last injection, in addition to blood collection, adrenal glands and visceral fat in the lower abdomen and pelvis were harvested from CRF-OE and WT mice and weighed to the nearest 0.1 mg.

### Experimental protocols

#### (1) Treatment with CRF receptor antagonists in CRF-OE mice with alopecia

The long-lasting peptide non-selective CRF receptor antagonist, astressin-B, the selective CRF_2_ receptor antagonist, astressin_2_-B [20], [22] or sterile isotonic saline was injected either ip or sc once a day for 5 days at 5 µg/0.1 ml/mouse in female and male CRF-OE mice (4–9 months old) with developed alopecia. The doses of astressin-B and astressin_2_-B were chosen based on our previous studies in mice to antagonize biological actions of ip CRF [22], [25]. Pigmentation and hair coverage scores were determined before the start of the first injection and at 1, 4 and 8 weeks (for ip injection experiments) and at 1, 2 and 4 weeks (for sc injection experiments) after the last injection.

The selective CRF_1_-non-peptide receptor antagonist, NBI-27914 (0.5 mg/mouse/injection) or its vehicle [1∶0.5∶8.5 (v/v) of ethanol, Tween-80 and saline, pH 5.0] was injected sc in female CRF-OE mice (5 months old) twice a day for 5 consecutive days. The regimen of NBI-27914 was chosen based on our previous studies [37]. Skin pigments and hair growth were monitored as in experiments with sc injection of astressin-B.

#### (2) Prevention of alopecia with CRF receptor antagonists in young CRF-OE mice with not yet developed alopecia

Young female CRF-OE mice (7–8 weeks old) that had not yet developed alopecia were injected sc with astressin-B, astressin_2_-B, or saline, as described above. Littermate WT mice served as control. Skin pigment and hair loss were monitored twice a week in the first 2 weeks following the initial injection, once a week up to 2 months, and monthly thereafter.

#### (3) Treatment of alopecia with minoxidil in CRF-OE mice

Female CRF-OE mice (5 months old) received a daily sc injection of 1% minoxidil sulfate (1 mg/mouse), or vehicle [1∶2∶7 (v/v) ethanol, polyethylene glycol and saline] for 10 consecutive days. The regimen of minoxidil administration was selected from previous studies showing facilitation of hair re-growth in another model of alopecia [38]. Skin pigment and hair growth were monitored as above following sc injection of astressin-B.

### Statistical analysis

Results are expressed as mean ± SEM or median. Comparisons of hair growth responses before and after treatment in the same animal were made using the before and after measure paired *t-test* while skin pigment scores were analyzed using Mann-Whitney rank sum test. Comparisons between different groups of hair growth responses as well as plasma corticosterone, adrenals and lower abdominal visceral fat weights were performed using either One Way ANOVA followed by Student-Newman-Keuls Method test or Student's *t-test* to compare two groups mean values. Differences in skin pigment between groups were compared using Kruskal-Wallis One Way Analysis of Variance on Ranks. P<0.05 was considered significant.
